# Effect of a Triterpenoid-Rich Olive Oil on Chronic Kidney Disease in an Experimental Model of Diabetes Mellitus

**DOI:** 10.3390/nu16162794

**Published:** 2024-08-21

**Authors:** José Pedro De La Cruz, Laura Osuna-Esteban, María Dolores Rodríguez-Pérez, Laura Ortega-Hombrados, Ana María Sánchez-Tévar, Esther Martín-Aurioles, María África Fernández-Prior, Sergio Pérez-Burillo, Juan Antonio Espejo-Calvo, José Antonio González-Correa

**Affiliations:** 1 Departamento de Farmacología, Instituto de Investigación Biomédica de Málaga y Plataforma en Nanomedicina—IBIMA Plataforma BIONAND, Facultad de Medicina, Universidad de Málaga, 29010 Malaga, Spain; jpcruz@uma.es (J.P.D.L.C.); lauraoc@uma.es (L.O.-E.); hombrados@uma.es (L.O.-H.); amstevar@uma.es (A.M.S.-T.); spburillo@uma.es (S.P.-B.); correa@uma.es (J.A.G.-C.); 2 Distrito Sanitario Málaga-Guadalhorce, UGC La Roca, 29009 Malaga, Spain; estherd.martin.sspa@juntadeandalucia.es; 3 Consejo Superior de Investigaciones Científicas (CSIC), Instituto de la Grasa, 41013 Sevilla, Spain; mfprior@ig.csic.es; 4 Tecnofood I+D+i Soluciones S.L., Instituto para la Calidad y Seguridad Alimentaria (ICSA), 18320 Granada, Spain; jaespejo@hotmail.com

**Keywords:** diabetes, nephropathy, olive oil, triterpenoids, oxidative stress, prostacyclin

## Abstract

The aim of this study was to assess the effect of triterpenoids on the development of diabetic nephropathy in an experimental model of diabetes mellitus. For this purpose, a destoned and dehydrated olive oil (DDOO) was used, comparing its effects to a destoned olive oil (DOO). DDOO had a higher triterpenoid content than DOO but an equal content of alcoholic polyphenols. Four study groups (*n* = 10 animals/group) were formed: healthy rats, diabetic control rats (DRs), and DRs treated orally with 0.5 mL/kg/day of DOO or DDOO for two months. DRs showed impaired renal function (proteinuria, increased serum creatinine, decreased renal creatinine clearance) and morphology (glomerular volume and glomerulosclerosis). These alterations correlated with increased systemic and renal tissue oxidative stress and decreased prostacyclin production. DDOO administration significantly reduced all variables of renal damage, as well as systemic and renal oxidative stress, to a greater extent than the effect produced by DOO. In conclusion, triterpenoid-rich olive oil may prevent kidney damage in experimental diabetes mellitus.

## 1. Introduction

Chronic kidney disease is one of the complications that appear in the evolution of diabetes mellitus, especially when glycemic control is inadequate [1,2]. More than 25–40% of patients with type 1 or type 2 diabetes mellitus suffer from nephropathy after 20–35 years of disease evolution [3]. The main clinical features of diabetic nephropathy are increased urinary albumin excretion (≥300 mg/day), decreased glomerular filtration rate, and progressive deterioration of renal function ultimately leading to end-stage renal failure [4]. The pathophysiology of diabetic nephropathy is complex and involves numerous biochemical pathways that are related to each other, mainly by enhancing one another. A state of sustained hyperglycemia activates, among others, four renal damage pathways: (1) nuclear activation of growth factor synthesis, favoring mesangial cell proliferation phenomena [5], (2) dysfunction of renal hemodynamics, mainly activating vasoconstrictor factor synthesis pathways [6], (3) activation of inflammatory pathways, inducing the synthesis of chemotactic molecules, leukocyte and endothelial adhesion molecules, nuclear synthesis factors of various interleukins, etc. [7], (4) induction of biochemical pathways that are set in motion to metabolize excess glucose (polyol, hexosamine, protein kinase C and glycation end-products pathways) [8]. The end products of each of these pathways can cause cell damage directly, by altering cell permeability, producing endothelial dysfunction, favoring cell proliferation, etc., but they also exert an indirect toxic effect, as they all produce oxidative stress, which in turn potentiates all the mechanisms of renal damage.

The generation of oxygen-derived free radicals (oxidative stress) and nitric oxide (nitrosative stress) can directly damage endothelial cells, podocytes, and mesangial cells [9,10]; on the other hand, oxygen-derived free radicals activate inflammatory, pro-fibrotic, and interstitial growth factor biochemical pathways [11], thus remodeling glomerular morphology and function; they also stimulate the production of vasoconstrictor mediators, such as thromboxane A_2_, and decrease that of vasodilator mediators, such as prostacyclin [12].

Based on the importance of oxidative stress in the evolution of diabetic nephropathy, the use of antioxidants has been postulated to prevent the appearance and/or evolution of this complication, without forgetting that glycemic control is the main preventive element of diabetes mellitus complications [13,14,15]. Extra virgin olive oil (EVOO) is a compound with a recognized high content of antioxidants, especially polyphenols; the administration of EVOO to patients with chronic kidney disease modifies several biomarkers related to this disease [16], observing a greater modification of renal function biomarkers when using an EVOO with high polyphenol content [17].

In an experimental model of diabetes mellitus in rats, used in the present study, the nephroprotective effect of hydroxytyrosol and 3’,4´-dihydroxyphenylyglycol [18,19], two polyphenols present in EVOO, has been confirmed. It has recently been postulated that triterpenoids of EVOO may have a beneficial effect on various cardiovascular conditions [20], which is why we proposed this study to try to find out the possible effect of these derivatives on diabetic nephropathy.

The aim of the present study is to evaluate the possible effect of an olive oil with a high content of triterpenoids, compared to another with the same polyphenol content but a lower concentration of triterpenoids, on various biomarkers of oxidative and nitrosative stress and renal function and morphology in an experimental model of diabetes mellitus.

## 2. Materials and Methods

### 2.1. Analytical Reagents

Colorimetric kits: thiobarbituric acid reactive substances (TBARS), total antioxidant capacity (TAC) (Cell Biolabs Inc., Bionova Científica S.L., Madrid, Spain), and reduced glutathione (Abcam, Cambridge, UK). Enzyme immunoassay kits: 3-nitrotyrosine, 8-iso-prostaglandin F2α (8-isoprostane), 8-hydroxy-2-deoxyguanosine (Cell Biolabs Inc., Bionova Científica S.L., Madrid, Spain), 11-dehydro-thromboxane B2, and 6-keto-prostaglandin F1α (Cayman Chemical Co., Ann Arbor, MI, USA). The rest of the reagents used were obtained from Sigma Chemical Corp. (St. Louis, MO, USA).

### 2.2. Olive Fruit Derived Products

Two types of oil were used, all of them from the Picual variety of olive, obtained and processed in the company Emilio Vallejo S.A. (Torredonjimeno, Jaen, Spain). The procedure followed from the harvesting of the Picual variety olives is as follows:

- Destoned olive oil (DOO)

It is obtained from Picual variety olives, which are destoned and subsequently subjected to a processing method using the two-stage cold extraction system [21]. The process includes separation of leaves and sticks, milling, beating, pressing of the paste obtained, washing with water, decanting, and obtaining the final olive oil. This procedure was carried out in the company Emilio Vallejo S.A. (Torredonjimeno, Jaen).

- Destoned and dehydrated olive oil (DDOO)

To obtain this oil, the olives are cleaned, stoned, and then dehydrated at a temperature not exceeding 40 °C. Finally, the dehydrated pulp is continuously centrifuged to obtain the final oil. The procedure is described by Olmo-García et al. [22]. The process comprises the following steps: (a) obtaining olive pulp; (b) dehydration of the olive pulp, resulting in dehydrated olive pulp; (c) milling of the dehydrated olive pulp, resulting in dry dehydrated olive powder; (d) obtaining olive oil from the dry dehydrated olive powder.

This procedure was carried out in the company Acer Camprestres S.L. (Castillo de Locubin, Jaén, Spain).

Table 1 shows the composition of the two types of oils administered to the experimental animals. The fatty acid composition of the oil was analyzed by simultaneous oil extraction and fatty acid methylation of the extracted oils. A gas–liquid chromatography (GLC) PerkinElmer Clarus 600 GC (PerkinElmer Inc., Waltham, MA, USA) was used. The GLC was equipped with a BPX70 30 m × 0.25 mm internal diameter × 0.25 µm film thickness capillary column (SGE Analytical Science Pty, Ltd., Ringwood, Australia). A split injector and flame ionization detector were maintained at 300 °C; a hydrogen was used as carrier gas (0.8 mL/min). Determination of aliphatic alcohols, sterols, and triterpenic dialcohols (erythrodiol and uvaol) was carried out in an Agilent 7890A gas chromatograph system (Agilent Technologies, Palo Alto, CA, USA) equipped with an FID detector. The analytical column was an HP-5 (5%-phenyl)-methylpolysiloxane column (30 m × 0.32 mm i.d., 0.25 μm film thickness).

### 2.3. Experimental Design

#### 2.3.1. Study Design, Care of the Animals, and Sample Size

Experimentation Center of the University of Malaga (CECA). The animals were included in the study when their weight was 250–270 g, and they underwent a quarantine period of one week at CECA. Afterward, they were individually identified and kept in individual cages until the end of the follow-up period, with food and drink ad libitum, recording the amounts of water and feed consumed by the animals each day.

This study was performed according to the guidelines of the Declaration of Helsinki, Law 14/2007 on Biomedical Research, and was approved by the Experimentation Ethics Committee of the University of Malaga (Ref. CEUMA31-2018-A) and the Consejería de Agricultura, Ganadería, Pesca y Desarrollo Sostenible, Junta de Andalucía (Ref. 9/07/2019/124). At all times, the animals were used in accordance with current Spanish legislation for the care, use, and housing of animals (EDL 2013/80847, BOE-A-2013-6271).

The recommendations of the Guide for the Care and Use of Laboratory Animals (NIH publication no. 86-23, revised 1985) were followed, as well as the Spanish Animal Protection Law, where applicable.

In the initial approach and throughout the experimental development, the 3R principles that must be complied with in all animal experimentation were observed:

- Replacement alternatives. The development of an experimental model of type 1 diabetes allows the reproduction of the macro- and microangiopathic complications that develop in patients with diabetes. This allows the evaluation of several biomarkers of vascular, cerebral, renal, and retinal damage, which enables the identification of potential therapeutic targets. This requires a living organism in which all the organ systems affected by chronic hyperglycemia are related, and this objective cannot be achieved in in vitro models.
- Reduction alternatives. The sample size was calculated based on a percentage difference in the main parameters that define diabetic nephropathy (proteinuria and glomerular volume) in treated diabetic animals with respect to untreated animals, calculating a percentage reduction of 30% for both parameters. The number of animals per group was 10, including a replacement rate of 10%.
- Refinement alternatives. All animals were handled by people with extensive experience in animal treatment and with official accreditation for handling experimental animals, and the suffering that could be generated by the procedures used was always reduced, minimizing the number of animal manipulations. Another way to minimize animal stress was to favor the environment in which the procedures were carried out. The animals were treated on a thermal blanket to maintain optimal body temperature, and noise sources were minimized. At the end of the treatment period, the animals were anesthetized with sodium pentobarbital, and after ensuring that the animal did not react to external stimuli, we proceeded to exsanguination and extraction of the organs under study. Subsequently, as this was a non-recovery procedure, the animals were decapitated with a guillotine, as established in the protocols for this type of experiment.

The following signs and symptoms were checked daily in the experimental animal:

- Presence of dyspnea or hemorrhage or stupor or cachexia was considered endpoint criteria.
- Presence of abnormal or increased secretions (no = 0 points; yes = 1 point); isolation or aggressive attitude towards conspecifics and/or investigator (no = 0 points; yes = 1 point); diarrhea (no = 0 points; yes = 1 point). In case of reaching 2 points, the endpoint criterion would have been applied.

No animal died during the experiment, and no endpoint criteria had to be applied.

#### 2.3.2. Groups of Study, Inclusion and Exclusion Criteria, and Randomization

Once the veterinarians of the Animal Experimentation Center of the University of Málaga confirmed that the experimental animals were healthy, they were randomly distributed into four groups, including them consecutively in the same order as described below (*n* = 10 rats per group):

- Group of normoglycemic control rats (NCR). In these animals, isotonic saline was administered in each of the procedures while the rest of the animals required the administration of some reagent or the study compounds.
- Group of diabetic control animals (DCR). These animals were induced with diabetes mellitus as explained in the following section, receiving saline instead of the study oils and 3–4 units of semi-slow-acting insulin (Levemir^®^, Novo Nordisk A/S, Bagsværd, Denmark) subcutaneously, to reduce the possibility of mortality due to excessive hyperglycemia.
- Group of diabetic animals treated with destoned olive oil (DOO) at a dose of 0.5 mL/kg/day orally.
- Group of diabetic animals treated with destoned and dehydrated olive oil (DDOO) at a dose of 0.5 mL/kg/day orally.

The distribution of each animal into the study groups was performed by a simple randomization system, not using randomization tables, but each animal randomly selected by one of the team members was included consecutively in order in the following groups: (1) healthy control group, (2) diabetic control group, (3) diabetic group treated with DOO, and 4) diabetic group treated with DDOO.

All animals were kept in individual cages, duly identified with a code, which was recorded in a workbook and in the computer database of the Animal Experimentation Center. The administration of saline (healthy control group), insulin, and the two types of oils used (diabetic rats) were always carried out by two members of the investigation team, always the same, confirming the identification of each animal by means of the code on the cage and in the workbook.

In all cases, the follow-up period was two months, and the administration of the oils or saline in the control groups was performed by orogastric cannulation in a single daily administration at 9:00 am. The administered dose of the oils was based on an adaptation of the administered dose of extra virgin olive oil in human studies (40–50 mL/day, which is equivalent to 0.5 mL/kg/day in persons of 75–80 kg body weight).

Diabetes mellitus was induced by a single dose of 40 mg/kg streptozotocin intraperitoneally. This experimental method is recognized as valid for inducing diabetes mellitus in rats like type 1 diabetes mellitus in humans. An animal was considered diabetic when two blood glucose determinations (tail vein) were obtained on two consecutive days, greater than 200 mg/dL (FreeStyle Glucometer. Laboratorios Abbot S.A., Madrid, Spain).

#### 2.3.3. Analytical Techniques

All analytical techniques were performed on all animals in this study, so each analytical determination was performed on 10 different samples for each experimental group. In no case was any sample excluded, and all of them were used for the analysis of the results.

##### Samples

On day 58, 24 h urine was collected in metabolic cages (Tecniplast S.p.A., Buguggiate, Italy). Samples were centrifuged at 3500× *g* for 10 min at 4 °C, dividing the sample into aliquots and freezing them at −80 °C.

On day 60 of follow-up, after a 12 h fasting period, the animals were anesthetized with sodium pentobarbital (40 mg/kg i.p.), and blood was drawn from the bifurcation of the iliac arteries. Subsequently, an isotonic saline solution was perfused at 37 °C. The animals were decapitated, and the following biological samples were obtained:

- Non-anticoagulated blood was centrifuged at 3500× *g* to obtain serum, obtaining aliquots, which were frozen at −80 °C.
- Renal tissue. The right kidney was processed for subsequent histological analysis, and the left kidney for determination of biochemical variables. From the left kidney, the renal cortex was homogenized in 50 mM phosphate-buffered saline, pH 7.0 (1/15 *w*/*v*), and then centrifuged at 13,000× *g* for 15 min at 4 °C. The supernatant was divided into aliquots and frozen at −80 °C.
- 24 h urine as described above.

##### Serum and Urinary Determination of Biochemical Variables

They were analyzed with the Atellica^®^CH autoanalyzer from Siemens Healthineers (Erlangen, Germany). Creatinine clearance was calculated according to the following standard formula [23]:

Creatinine clearance = urine creatinine (mg/dL) × urine volume (mL)/serum creatinine (mg/dL) × 1000/body weight (g) × 1/1440.

##### Oxidative and Nitrosative Stress

As an index of lipid peroxide concentration in serum and renal tissue, the determination of substances reactive to thiobarbituric acid was quantified, as malondialdehyde is the main product of this reaction. We determined urinary 8-isoprostane concentration for global quantification of oxidative stress [24]. As an index of oxidative stress-induced DNA damage, 8-hydroxy-2-deoxyguanosine was quantified. Antioxidant defense was measured using serum and renal glutathione concentrations, glutathione peroxidase activity, and total antioxidant capacity. As an index of nitrosative stress (peroxynitrite formation), we measured 3-nitrotyrosine concentrations in serum and kidney tissue. All these parameters were measured according to the manufactured protocol.

##### Urinary Prostanoids

Thromboxane and prostacyclin production was quantified by determining urinary concentrations of their main metabolites 11-dehydrothromboxane B_2_ and 6-keto-prostaglandin F_1α_, respectively, according to the manufacturer protocol.

##### Morphometric Analysis

Kidney was fixed in 10% formalin, obtaining 5 μm sections, and stained with hematoxylin and eosin and periodic acid Schiff (PAS) according to rutinary laboratory methods. The stained sections were analyzed with an image analysis system (Olympus BX-UCB, with VS-ASW FL software, Olympus, Hamburg, Germany). QuPach-0 program and the FIJI ImageJ program (https://imagej.net/, accessed on 3 October 2023) were used to perform quantitative analysis. Glomerular volume (GV) was calculated according to Lane et al. [25].

GV = (GA)3/2 × β/d

where GA is the glomerular area, β is a dimensionless shape coefficient (β = 1.0 for perfect spheres), and d is a size distribution coefficient that adjusts for variations in glomerular size.

To calculate the glomerulosclerosis rate (GMR), PAS-stained sections (50 glomeruli/section) were used. After assessing the area of each glomerulus, the PAS-positive surface area was quantified by image analysis using the following formula:

[PAS(+)A (μm^2^)/GA (μm^2^)] × 100

where GMR is the percentage of glomerular area with PAS(+) stained surface; PAS(+)A: area occupied with PAS(+) material in a glomerulus; GA: area of a glomerulus.

### 2.4. Statistical Analysis

Univariate analysis: The data in the text, tables, and figures represent the mean ± standard deviation of 10 animals per experimental group. All statistical analyses were performed with the statistical package for social sciences version 29.0 (SPSS Co., Chicago, IL, USA) licensed to the Central Computer Service of the University of Malaga. Bivariate analysis was performed using ANOVA with a subsequent Bonferroni transformation to evaluate differences between treatment groups. Student’s *t*-test for unpaired data was used to establish the difference between the variables analyzed in the non-diabetic control group vs. diabetic control group. The biochemical–morphological correlation was established by calculating Pearson’s correlation coefficients. Statistical significance was considered for a *p*-value of less than 0.05.

## 3. Results

### 3.1. Zoometric Variables

Diabetic animals increased their body weight significantly less than non-diabetic animals. Diabetic animals treated with both types of oils showed significantly higher values than diabetic controls. Kidney weights were 66% higher in diabetic animals, with a 33% increase in animals treated with both types of oils (Table 2).

Diabetic animals ingested 34% less food and drank 3.4 times more water than normoglycemic animals. Food intake was significantly increased with both types of oils, and water intake was significantly reduced, although the values of the healthy group of animals were not reached (Table 2).

### 3.2. Serum and Urinary Biochemistry

The induction of the diabetes mellitus model significantly elevated blood glucose levels, which were reduced in the animals treated with oil from destoned and dehydrated olives, although significantly elevated blood glucose levels were maintained with respect to healthy animals. The same behavioral profile was observed with urine glucose values (Table 3).

Serum creatinine was significantly higher in diabetic control animals (66% with respect to healthy controls), and urinary creatinine was 41.5% lower in urine. The administration of both types of oils reduced serum creatinine levels by 20% and increased urinary creatinine concentration by 17% (destoned olive oil) and 27% (destoned and dehydrated olive oil) (Table 3).

No statistically significant differences in serum protein levels were observed in any of the study groups, however, urine protein concentration was significantly increased, decreasing by 43.5% after treatment with destoned olive oil and by 81% with destoned and dehydrated olive oil (Table 3).

The lipid profile of diabetic control animals was characterized by a significant increase in serum levels of total cholesterol, LDL cholesterol, and triglycerides, as well as a decrease in serum HDL cholesterol concentration. Administration of both types of oils significantly reduced the increased parameters and elevated those of HDL cholesterol; except for the effect on HDL cholesterol concentration, administration of destoned and dehydrated olive oil produced a significantly greater effect than destoned olive oil (Table 3).

The urinary excretion of prostanoids was significantly different in diabetic control animals, that of the thromboxane metabolite being 2.8 times higher and that of the prostacyclin metabolite being 62% lower. The administration of both types of oils reduced the excretion of the thromboxane metabolite and increased that of the prostacyclin metabolite, these values being normalized with oil from destoned and dehydrated olives (Table 3).

### 3.3. Kidney Variables

**Figure 1:**
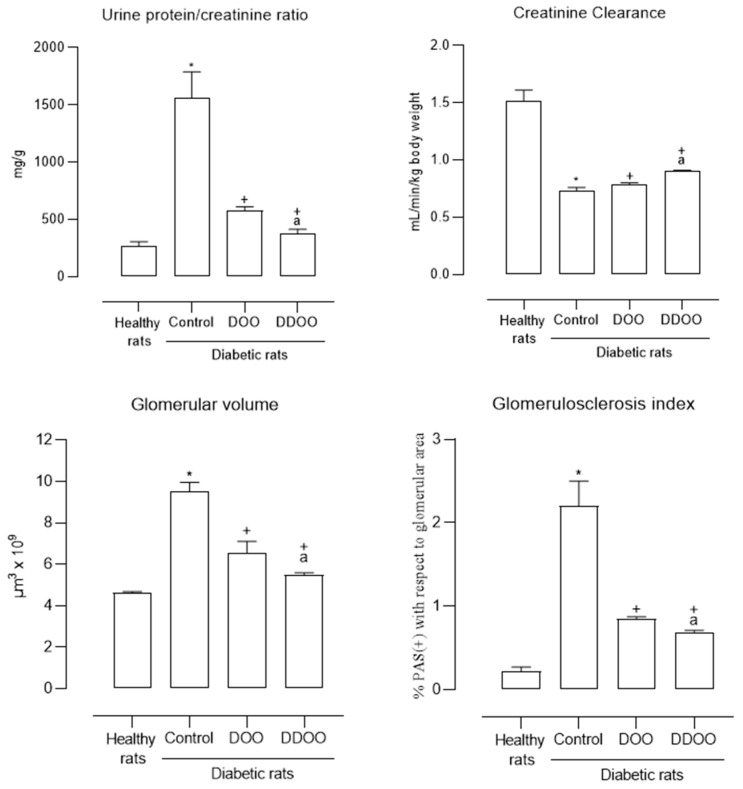
Mean values (mean ± standard deviation) of biochemical (urine protein/creatinine ratio and creatinine clearance) and morphological (glomerular volume and glomerulosclerosis index) kidney variables from healthy rats, control diabetic rats, and diabetic rats treated with 0.5 mL/kg/day p.o. of destoned olive oil (DOO) or destoned and dehydrated olive oil (DDOO). *n* = 10 rats/group. * *p* < 0.05 with respect to healthy rats, ^+^ *p* < 0.05 with respect to control diabetic rats, ^a^ *p* < 0.05 with respect to DOO.

Urinary protein excretion, in relation to urinary creatinine concentration, was significantly increased in diabetic control animals, and this ratio was reduced in diabetic animals treated with both types of oil, although those to which destoned and dehydrated olive oil was administered showed significantly lower values (75.5% reduction compared to 62.5% with destoned olive oil) (Figure 1).

Renal creatinine clearance was reduced by 52% in diabetic control animals, increasing by 12.3% after administration of destoned olive oil and by 21.9% with destoned and dried olive oil (Figure 1).

**Figure 2:**
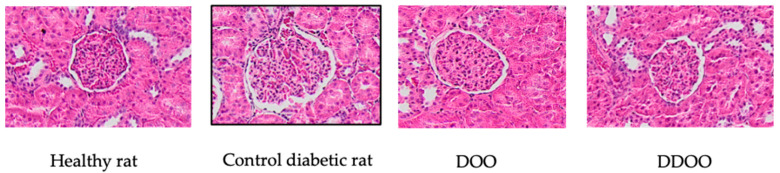
Representative examples of glomerular images from kidneys of a healthy rat, a control diabetic rat, and diabetic rats treated with 0.5 mL/kg/day p.o. of destoned olive oil (DOO) or destoned and dehydrated olive oil (DDOO). Stain: haematoxylin-eosin (X20). This staining shows that the glomerulus of the diabetic control animal is larger than the others and that treatment with DOO and DDOO reduces the glomerular area, without reaching the size of the glomerulus of the healthy control animal.

**Figure 3:**
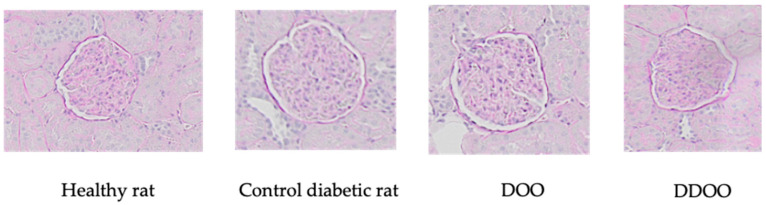
Representative examples of PAS-stained glomerulus (X20) from kidneys of a healthy rat, a control diabetic rat, and diabetic rats treated with 0.5 mL/kg/day p.o. of destoned olive oil (DOO) or destoned and dehydrated olive oil (DDOO). These images show expansion of mesangium and accumulation of PAS-positive material in control diabetic rat.

Morphological analysis of renal samples showed a significant increase in glomerular volume (2.1 times higher) and glomerulosclerosis index (10 times higher). Both types of oils reduced both parameters: oil from destoned olives by 31% the glomerular volume and 61% the glomerulosclerosis index, oil from destoned and dehydrated olives by 40% the glomerular volume and 68% the glomerulosclerosis index (Figure 1). Figure 2 and Figure 3 show some representative examples of these histological determinations.

### 3.4. Oxidative and Nitrosative Stress

Diabetic control animals showed a significant increase in all variables related to oxidative and nitrosative stress, increasing those indicating an excess of oxidative processes and a reduction of antioxidant defense parameters, both in serum and renal tissue samples. Administration of destoned olive oil decreased serum and renal oxidative and nitrosative stress. The administration of destoned and dehydrated olive oil also reduced the concentrations of oxidative and nitrosative stress variables, its effect being significantly greater than that shown by destoned olive oil in all variables except total antioxidant capacity in renal tissue (Table 4).

### 3.5. Correlation Analysis

Kidney variables such as urine protein/creatinine ratio, creatinine clearance, glomerular volume, and glomerulosclerosis index correlated statistically with oxidative and nitrosative stress, both in serum and in kidney, and prostanoid production (Table 5).

## 4. Discussion

The results obtained in this study demonstrate that the administration of destoned and dehydrated olive oil, rich in triterpenoids and equal in total polyphenol content to destoned olive oil, has a greater nephroprotective and antioxidant effect in an experimental model of diabetes mellitus like human type 1 diabetes.

Due to the importance of free radicals and oxidative stress in the development and evolution of chronic kidney disease in diabetes, the administration of antioxidant compounds has been proposed as an intervention that could help the effect that strict glycemic control has in the delay and/or prevention of macro- and microangiopathic complications of diabetes mellitus [26]. The results obtained in this study show that in the experimental model used, there is clear oxidative stress, both systemic (serum determinations) and in the renal tissue itself (Table 4), thus ratifying this important pathophysiological aspect. Likewise, a statistically significant relationship has been determined between variables of renal damage (morphological and functional) and the variables that define oxidative stress, both systemic and renal (Table 5).

Several studies demonstrate the effect of certain natural antioxidants in the prevention of chronic kidney disease, mainly on dietary polyphenol composition. Some polyphenols have shown a renal protective effect in experimental models of ischemia–reperfusion in relation to their antioxidant and anti-inflammatory effects, such as the case of resveratrol, gallic acid, epigallocatechin-3-gallate, ellagic acid, curcumin, among others. Some polyphenols have shown a protective effect in diabetic nephropathy models, such as resveratrol [27], polydatin, cyanidin 3-glucoside, epicatechin, oligonol, among others [28].

With respect to the polyphenols in extra virgin olive oil, as part of the Mediterranean-type diet, it has been shown that oleuropein, as well as hydroxytyrosol and 3′,4′-dihydroxyphenylglycol, have a nephroprotective effect in the same experimental model used in this study, always in relation to their antioxidant capacity [18,19,29]. Other compounds present in extra virgin olive oil are triterpenoids, such as oleanolic acid and maslinic acid, both with important antioxidant and nephroprotective effects in experimental diabetes mellitus models, probably through activation of the NRF2 pathway [30].

We cannot rule out the idea that in extra virgin olive oil, all these polyphenols are present and could exert a synergistic effect among them, as has been demonstrated for hydroxytyrosol and 3′,4′-dihydroxyphenylglycol at the level of cardiovascular biomarkers and neuroprotection in experimental type 1 diabetes mellitus [31,32] and in platelet aggregation [33]. For this reason, to study the possible effect of triterpenoids in the diet, it is necessary to consider the effect of the other polyphenols present in extra virgin olive oil, which is why we have used an olive oil containing the same total content in alcoholic polyphenols, whose nephroprotective effect has been demonstrated, but one of those oils with a high content in triterpenoids (DDOO).

First, it should be noted that there are effects on non-renal biomarkers in the experimental model of diabetes, which may influence the development of diabetic nephropathy, such as hyperlipemia status (Table 3) or systemic oxidative stress (Table 4); in both cases, DDOO showed greater effect than DOO. That is, although alcoholic polyphenols exert a beneficial effect at this level, the presence of triterpene derivatives enhances this effect. Studies on the hypolipidemic effect of hydroxytyrosol refer to models of type 2 diabetes [34], while those with oleanolic acid show a hypolipidemic effect in models of type 1 diabetes [35], as we have shown in this study.

Regarding the antioxidant effect, it is known that alcoholic polyphenols, such as hydroxytyrosol, have a clear antioxidant effect in models of diabetes mellitus and in various organs, such as the brain and kidney, as well as at the systemic level in blood serum [19,36]. Regarding triterpenoid derivatives, a study in healthy volunteers administered an extra virgin olive oil enriched with oleanolic and maslinic acid shows a greater effect on urinary elimination of 8-isoprostanes and 8-hydroxy-2-deoxyguanosine compared to an extra virgin olive oil with a lower triterpenoid content [37]. This study coincides with the results obtained in our experimental model of diabetes mellitus as far as the antioxidant effect is concerned. The antioxidant effect of oleanolic acid and maslinic acid has been related to an anti-inflammatory and cytoprotective effect at the cerebral and cardiac levels [38,39,40,41].

Considering the importance of oxidative stress in the development of diabetic nephropathy, the oils used in this study should improve renal function in diabetic animals due to the content of alcoholic polyphenols present in their composition, and olive oil from destoned and dehydrated olives should present a greater effect at this level due to its higher content of triterpenoids. Indeed, the animals treated with destoned olive oil reduced the variables that indicate morphological and functional damage to the kidneys of diabetic animals, this nephroprotective effect being greater in the animals treated with destoned and dehydrated olive oil (Table 3 and Figure 1).

In addition, an imbalance in the thromboxane/prostacyclin ratio was also observed, in favor of the vasoconstrictor compound, a fact that could also participate in the development of diabetic nephropathy. The administration of destoned olive oil decreased the urinary excretion of the thromboxane metabolite and reduced the inhibition of prostacyclin production, this effect being greater in the triterpenoid-rich oil group. Hydroxytyrosol has shown an inhibitory effect on cyclooxygenase, thus reducing platelet and renal thromboxane production, and its antioxidant effect decreases the degradation of prostacyclin by free radicals [36]. Regarding triterpenoids, it has been shown that oleanolic acid induces prostacyclin production in vascular smooth muscle cell cultures in relation to a mechanism related to type-2 cyclooxygenase [42], and maslinic acid inhibits platelet aggregation induced by platelet thromboxane [43].

Finally, the importance of these mechanisms is reflected in the existence of a correlation (Table 5) between the main variables of renal damage and the rest of the biochemical variables studied, a fact that had already been demonstrated for hydroxytyrosol and for 3′,4′-dihydroxyphenylglycol [18,19].

Despite these results, this study has some limitations. Firstly, we have focused on the explanation of the nephroprotective effect of DDOO on the greater presence of triterpenoids since DOO showed a nephroprotective effect but quantitatively less. These results do not indicate that triterpenoids are the only ones responsible for the nephroprotective effect since, as mentioned above, alcoholic polyphenols have already demonstrated this effect; however, it cannot be ruled out that other compounds in extra virgin olive oil may also exert a nephroprotective effect, although this has not yet been reported in the literature. The main limitation of this study at this level is that we have not performed experiments using these compounds, alone or in combination, so we must assume that the triterpenoids have a joint nephroprotective effect with the rest of the polyphenols either through an additive or potentiation mechanism. Future association studies of alcoholic polyphenols and triterpenoids are needed to assess the possible positive interaction between them, as well as the true role of triterpenoids on the mechanisms of diabetic nephropathy. Another limitation of this study was the use of a simple randomization system, not using any randomization generator system, as used in human studies. Furthermore, this study has been performed in an experimental model like human type 1 diabetes, so a future study in an experimental model of type 2 diabetes is needed. Both types of studies are currently at an early stage in our laboratory.

## 5. Conclusions

The administration of an olive oil rich in triterpenoids, in the presence of the rest of the polyphenols present in extra virgin olive oil, shows a greater nephroprotective effect in an experimental model of diabetes type 1, relating this effect to an antioxidant mechanism, as well as an effect on the prostanoids thromboxane and prostacyclin.

## Figures and Tables

**Table 1:** Composition of destoned olive oil (DOO), and destoned and dehydrated olive oil (DDOO).

Parameter	Destoned and Dehydrated Olive Oil (DDOO)	Destoned Olive Oil (DOO)
Acidity (%)	0.11 ± 0.01	0.11 ± 0.01
Peroxide value (meqO_2_/kg)	10.22 ± 1.12	9.05 ± 0.0
K270	0.15 ± 0.01	0.16 ± 0.01
K232	1.55 ± 0.03	1.74 ± 0.03
Delta K	<0.01	<0.01
Ethyl esters (mg/kg)	12 ± 2	14 ± 2
Ethyl palmitate (mg/kg)	4 ± 0	6 ± 1
Ethyl oleate (mg/kg)	6 ± 1	7 ± 1
Waxes (mg/kg)	45 ± 2	32 ± 1
Fatty acid composition		
Myristic acid (%)	0.01 ± 0.00	0.01 ± 0.00
Palmitic acid (%)	12.92 ± 1.11	11.58 ± 0.79
Palmitoleic acid (%)	1.22 ± 0.04	1.1 ± 0.05
Margaric acid (%)	0.05 ± 0.00	0.05 ± 0.00
Margaroleic acid (%)	0.08 ± 0.00	0.07 ± 0.00
Stearic Acid (%)	2.48 ± 0.71	2.95 ± 0.55
Oleic Acid (%)	78.05 ± 3.54	77.59 ± 2.98
Linoleic Acid (%)	4.01 ± 0.55	4.52 ± 0.72
Linolenic Acid (%)	1.02 ± 0.04	0.91 ± 0.03
Arachidic Acid (%)	0.35 ± 0.01	0.32 ± 0.01
Eicosanoic Acid (%)	0.15 ± 0.01	0.21 ± 0.01
Bhenenic Acid (%)	0.12 ± 0.01	0.15 ± 0.01
Lignoceric acid (%)	0.09 ± 0.01	0.07 ± 0.00
Total sterols (mg/kg)	1428 ± 18	1227 ± 12
Cholesterol (%)	0.1 ± 0.0	0.1 ± 0.0
Brassicasterol (%)	<0.1	<0.1
Cholesterol (%)	2.4 ± 0.1	2.9 ± 0.0
Stigmasterol (%)	0.9 ± 0.1	0.5 ± 0.0
B-Sitosterol (%)	96.4 ± 3.2	93.2 ± 3.0
D7-Stigmastenol (%)	0.3 ± 0.0	0
Erythrodiol + Uvaol (%)	3.6 ± 0.1	0.2 ± 0.0
Total triterpenic acids (mg/kg)	845.21 ± 16.80	122.1 ± 5.07
Oleanolic acid (mg/kg)	261.04 ± 10.84	37.59 ± 2.12
Maslinic acid (mg/Kg)	574.72 ± 14.01	80.53 ± 2.30
Ursolic acid (mg/kg)	9.45 ± 0.10	4.02 ± 0.09
Chlorophyll Pigments (mg/kg)	32.84 ± 1.02	31.66 ± 1.58
Carotenoid Pigments (mg/kg)	7.44 ± 0.23	8.95 ± 0.17
Squalane (mg/100 g)	594.32 ± 24.51	388.05 ± 32.87
Tocoferoles (mg/kg)	421.16 ± 12.75	388.04 ± 14.60
Total phenols (ppm)	752.15 ± 23.15	722.61 ± 20.07
3,4-dihydroxyphenylglycol	1.32 ± 0.02	1.17 ± 0.02
Hydroxytyrosol	12.14 ± 0.44	8.01 ± 0.22
Tyrosol	12.07 ± 1.33	4.52 ± 1.74
Vanillic acid	0	0
HT acetate	14.1 5 ± 0.55	0
Nuzhenide	7.12 ± 0.75	0
Oleuropein derivative 1	0	0
Oleuropein derivative 2	28.74 ± 2.21	52.94 ± 4.50
Ligustroside derivative	57.09 ± 3.18	62.15 ± 2.33
Sum	132.63 ± 7.52	128.91 ± 8.45
Hydroxytyrosol (HT) potential (ppm)	33.05	27.60
% of potential HT approx.	0.0033	0.0028

There are no statistical differences, except for total triterpenic acids (*p* < 0.05).

**Table 2:** Zoometric variables (mean ± standard deviation) after two months follow-up in control healthy rats, control diabetic rats, and diabetic rats treated with 0.5 mL/kg/day p.o. of destoned olive oil and destoned and dehydrated olive oil. *n* = 10 rats per group.

	Control Healthy Rats	Control Diabetic Rats (DRs)	DRs Treated with Destoned Olive Oil	DRs Treated with Destoned and Dehydrated Olive Oil
Body weight (g)				
Day 1	298 ± 9.2	286 ± 10.1	290 ± 8.3	293 ± 9.0
Day 60	505 ± 8.3	380 ± 19.1 *	419 ± 35.0 ^+^	397 ± 33.1 ^+^
% increase	157 ± 10.6	134 ± 17.5 *	149 ± 21.3 ^+^	141 ± 29.9 ^+^
Kidney weight (% respect to body weight)	0.6 ± 0.05	1.0 ± 0.07 *	0.8 ± 0.06 ^+^	0.8 ± 0.05 ^+^
Chow consumption (g/day)	32.4 ± 5.1	21.4 ± 2.3 *	23.8 ± 3.3 ^+^	27.2 ± 5.2 ^+^
Drinking water (mL/day)	43.3 ± 16.5	145 ± 27.5 *	90.5 ± 17.0 ^+^	90.6 ± 15.5 ^+^

* *p* < 0.05 with respect to control healthy rats. ^+^ *p* < 0.05 with respect to control diabetic rats.

**Table 3:** Biochemical variables (mean ± standard deviation) after two months follow-up in control healthy rats, control diabetic rats, and diabetic rats treated with 0.5 mL/kg/day p.o. of destoned olive oil and destoned and dehydrated olive oil. *n* = 10 rats per group.

	Control Healthy Rats	Control Diabetic Rats (DRs)	DRs Treated with Destoned Olive Oil	DRs Treated with Destoned and Dehydrated Olive Oil
Serum				
Glucose (mg/dL)	98.8 ± 6.0	531 ± 10.1 *	508 ± 13.1 ^+^	435 ± 9.9 ^+a^
Creatinine (mg/dL)	0.6 ± 0.05	1.0 ± 0.07 *	0.8 ± 0.06 ^+^	0.8 ± 0.05 ^+^
Total proteins (g/dL)	6.3 ± 0.1	6.1 ± 0.3	6.2 ± 0.3	5.8 ± 0.2
Total cholesterol (mg/dL)	53.4 ± 3.5	86.4 ± 4.0 *	70.6 ± 2.1 ^+^	63.2 ± 3.7 ^+a^
LDL cholesterol (mg/dL)	21.4 ± 1.4	44.8 ± 4.7 *	26.7 ± 5.1 ^+^	17.3 ± 2.7 ^+a^
HDL cholesterol (mg/dL)	18.6 ± 1.5	15.3 ± 0.8 *	27.9 ± 3.4 ^+^	32.1 ± 1.4 ^+^
Triglycerides (mg/dL)	56.7 ± 7.7	149 ± 8.0 *	106 ± 3.5 ^+^	85.5 ± 5.1 ^+a^
Urine Creatinine (mg/dL)	114 ± 8.3	66.6 ± 3.5 *	78.1 ± 5.6 ^+^	85.1 ± 4.2 ^+^
Proteinuria (mg/L)	14.8 ± 1.2	100 ± 10.3 *	56.5 ± 5.7 ^+^	18.5 ± 1.7 ^+a^
Glucosuria (g/L)	0.0 ± 0.0	7.3 ± 0.7 *	6.2 ± 0.6 ^+^	4.8 ± 0.4 ^+a^
11-dH-TxB2 (ng/mg creatinine)	4.7 ± 0.6	13.2 ± 0.6 *	8.8 ± 0.6 ^+^	5.1 ± 0.4 ^+a^
6-keto-PGF1α (pg/mg creatinine)	19.5 ± 0.3	7.4 ± 0.6 *	13.2 ± 0.4 ^+^	17.7 ± 0.7 ^+a^

11-dH-TxB_2_: 11-dehydro-thromboxane B_2_. 6-keto-PGF_1α_: 6-keto-prostaglandin F_1α_. * *p* < 0.05 with respect to control healthy rats. ^+^ *p* < 0.05 with respect to control diabetic rats. ^a^ *p* < 0.05 with respect to diabetic rats treated with destoned olive oil.

**Table 4:** Oxidative stress variables (mean ± standard deviation) after two months follow-up in control healthy rats, control diabetic rats, and diabetic rats treated with 0.5 mL/kg/day p.o. of destoned olive oil and destoned and dehydrated olive oil. *n* = 10 rats per group.

	Control Healthy Rats	Control Diabetic Rats (DRs)	DRs Treated with Destoned Olive Oil	DRs Treated with Destoned and Dehydrated Olive Oil
Serum				
TBARS (nmol/mL)	4.6 ± 0.9	10.1 ± 0.6 *	4.2 ± 1.1 ^+^	1.7 ± 0.2 ^+a^
8-OhdG (ng/mL)	17.7 ± 0.5	29.0 ± 1.8 *	4.5 ± 0.2 ^+^	1.5 ± 0.2 ^+a^
GSH (nmol/mL)	139 ± 8.6	90.2 ± 0.9 *	106 ± 3.5 ^+^	139 ± 1.0 ^+a^
GSHpx (nmol/min/mL)	30.7 ± 1.2	8.0 ± 0.7 *	18.5 ± 0.9 ^+^	27.7 ± 2.4 ^+a^
TAC (IU/mL)	19.6 ± 0.6	14.6 ± 0.8 *	17.3 ± 1.0 ^+^	18.7 ± 0.6 ^+a^
3-nitrotyrosine (pg/mL)	16.2 ± 1.1	73.3 ± 2.1 *	49.1 ± 1.4 ^+^	42.9 ± 1.4 ^+a^
Kidney				
TBARS (nmol/mg protein)	3.8 ± 0.5	24.0 ± 0.8 *	15.8 ± 0.2 ^+^	14.2 ± 0.1 ^+a^
8-isoprostane (ng/mg creatinine)	7.4 ± 0.5	53.9 ± 0.7 *	16.4 ± 0.6 ^+^	14.6 ± 0.5 ^+a^
8-OHdG (ng/0.1 g tissue)	7.2 ± 0.8	13.8 ± 0.7 *	9.7 ± 0.1 ^+^	8.3 ± 0.3 ^+a^
GSH (µmol/0.1 g tissue)	521 ± 25.0	129 ± 13.6 *	187 ± 3.1 ^+^	291 ± 7.1 ^+a^
GSHpx (nmol/min/0.1 g tissue)	105 ± 5.3	59.0 ± 2.1 *	89.8 ± 3.3 ^+^	98.2 ± 1.5 ^+a^
TAC (IU/0.1 g tissue)	104 ± 2.9	35.7 ± 3.2 *	83.5 ± 1.5 ^+^	97.7 ± 3.1 ^+^
3-nitrotyrosine (pg/0.1 g tissue)	22.7 ± 1.5	131 ± 6.9 *	74.1 ± 1.6 ^+^	59.7 ± 4.1 ^+a^

TBARS: thiobarbituric acid reactive substances. 8-OHdG: 8-di-hydroxy-deoxy guanosine. GSH: reduced glutathione. GSHpx: glutathione peroxidase activity. TAC: total antioxidant capacity. * *p* < 0.05 with respect to control healthy rats. ^+^ *p* < 0.05 with respect to control diabetic rats. ^a^ *p* < 0.05 with respect to diabetic rats treated with destoned olive oil.

**Table 5:** Pearson correlation coefficients between renal function variables and biochemical variables analyzed.

	Prot/Creat	CrCl	GV	GlS
Prot/Creat	----	−0.946 *	0.898 *	0.968 *
CrCl	−0.946 *	----	−0.798 **	−0.942 *
GV	0.898 *	−0.798 **	----	0.879 *
GlS	0.968 *	−0.942 *	0.879 *	----
TBARS (s)	0.932 *	−0.881 *	0.903 *	0.918 *
8-OH-dG (s)	0.973 *	−0.958 *	0.855 *	0.984 *
3-Nty (s)	0.877 *	−0.768 ***	0.861 *	0.832 *
8-isoprostanes	0.929 *	−0.852 *	0.864 *	0.924 *
TAC (s)	−0.873 *	0.807 **	−0.839 *	−0.827 *
GSH (s)	−0.891 *	0.805 **	−0.785 **	−0.875 *
GSHpx (s)	−0.917 *	0.840 *	−0.835 *	−0.935 *
TBARS (k)	0.916 *	−0.969 *	0.815 *	0.946 *
8-OH-dG (k)	0.937 *	−0.917 *	0.769 ****	0.915 *
3-Nty (k)	0.952 *	−0.918 *	0.871 *	0.948 *
TAC (k)	−0.950 *	0.896 *	−0.862 *	−0.936 *
GSH (k)	−0.945 *	0.872 *	−0.869 *	−0.934 *
GSHpx (k)	−0.910 *	0.947 *	−0.744 *****	−0.935 *
TxB_2_	0.971 *	−0.885 *	0.881 *	0.965 *
PGI_2_	−0.967 *	0.939 *	−0.850 *	−0.963 *

* *p* < 0.001, ** *p* < 0.002. *** *p* < 0.004, **** *p* < 0.003, ***** *p* < 0.006. 3-NTy: 3-nitrotyrosine. 8-OH-dG: 8-hydroxy-2-deoxyguanosine. CrCl: creatinine clearance. GlS: glomerulosclerosis index. GSH: reduced glutathione. GSHpx: glutathione peroxidase activity. PGI_2_: urine 6-keto-PGF_1α_. Prot/Creat: urine protein/creatinine ratio. TAC: Total antioxidant capacity. TBARS: thiobarbituric acid reactive substances. TxB_2_: urine 11-dinor-thromboxane B_2_. GV: glomerular volume. (s): serum. (k): kidney.

## Data Availability

The data presented in this study are available in the article.
